# The conceptualisation of patient-centred care: A case study of diabetes management in public facilities in southern Malawi

**DOI:** 10.4102/phcfm.v13i1.2755

**Published:** 2021-09-20

**Authors:** Martha Makwero, Adamson Muula, Felix C. Anyanwu, Jude Igumbor

**Affiliations:** 1 Department of Family Medicine, School of Public Health and Family Medicine, University of Malawi, Blantyre, Malawi; 2 Department of Public Health, School of Public Health, University of the Witwatersrand, Johannesburg, South Africa; 3 Department of Public Health, School of Public Health and Family Medicine, University of Malawi, Blantyre, Malawi; 4 Department of Public Health, School of Health Sciences, University of Venda, Limpopo, South Africa; 5 Faculty of Health Sciences, Dora Ngiza Provincial Hospital, Port Elizabeth, South Africa

**Keywords:** patient-centred care, diabetes mellitus, conceptualisation, elements perceptions, chronic care, quality of care, patient involvement

## Abstract

**Background:**

Patient-centred care (PCC) is one of the pillars of Malawi’s quality of care policy initiatives. The role of PCC in facilitating quality service delivery is well documented, and its importance may heighten in chronic disease management. Yet, PCC conceptualisation is known to be context specific.

**Aim:**

The study aimed to understand the conceptualisation of PCC amongst patients, healthcare providers (HCP) and policy makers in Diabetes Mellitus (DM) management.

**Setting:**

This study was conducted in DM clinics in Southern Malawi.

**Methods:**

Our qualitative exploratory research study design used in-depth and focus group interviews. We interviewed patients with DM, HCPs and policy makers. The study used framework analysis guided by Mead and Bower’s work.

**Results:**

Patient-centred care conceptualisations from groups of participants showed convergence. However, they differed in emphasis in some elements. The prominent themes emerging from the participants’ conceptualisation of PCC included the following: meeting individual needs, goals and expectations, accessing medication, supporting relationship building, patient involvement, information sharing, holistic care, timeliness and being realistic.

**Conclusion:**

Patient-centred care conceptualisation in Malawi goes beyond the patient–HCP relational framework to include the technical aspects of care. Contrary to the global view, accessing medication and timeliness are major elements in PCC conceptualisation in Malawi. Whilst PCC conceptualisation is contextual, meeting expectations and needs of patients is fundamental.

## Background

The shifting global disease burden from acute to chronic, and the associated multi-morbidity, has necessitated that care delivery moves away from the traditional acute, episodic and biomedical models to embrace more patient-centred care (PCC) values.^1,2^ The World Health Organization (WHO) innovations for chronic care advance PCC delivery as an approach that helps patient-healthcare provider (HCP) encounters to deal with the complex bio-psychosocial determinants of health and enhance care experiences whilst meeting the needs and expectations of people seeking care.^3^

In Malawi, the chronic disease burden is growing with Diabetes Mellitus (DM) disease prevalence now at 3.0% and 1.7% for urban and rural areas, respectively, and accounts for 2.4% of the Daily Adjusted Life Years (DALYs).^4^ Notably, DM care in Malawi is reportedly suboptimal as appraised by Assayed et al. in Malawi district using International Diabetes Federation (IDF) guidelines whose notable feature is the organisation of care around the patient.^5,6^ Consequently, the Malawi government promotes the PCC approach in chronic disease management.^7^ However, PCC conceptualisation is not homogenous across contexts making it difficult to objectively appraise its performance.

Markedly, the success of PCC implementation and its appraisal lies in the clarity of its functional elements. For example, in the National Health-Care quality report by the Agency for Health Research and Quality (AHRQ), PCC showed a modest improvement of 1.9% increase in its contribution to the overall improvement in quality of care, lagging behind patient safety (10.3%) and efficiency (3.0%).^8^ The lag was largely related to the vagueness of what constitutes PCC, which thwarted its implementation and appraisal. Most African literature offers no specific conceptualisation. However, some elements such as ‘people first’, accountability to the patient, holism, communication, HCP attitudes of friendliness, respect and empathy and family involvement do emerge in describing PCC.^9,10,11^ This makes PCC conceptualisation blurry and hampers its successful implementation.

Literature records variations in PCC conceptualisation, largely depending on context and whose perspective is being represented; whether patients or HCPs.^12,13,14,15^ Conceptualisation refers to the process of breaking down concepts into common functional meanings.^16^ The Institute of Medicine (IOM) conceptualises PCC as care that is responsive to individual patient preferences, needs and values, ensuring that patient wishes guide all clinical decisions.^17^ The reliance on patients’ perspectives, values and wishes to guide all clinical decisions may appear consumeristic and would promote unhealthy patients’ agenda, falling short of scientifically sound patient care decisions. Marshall et al. advise that good conceptualisations should be co-constructed using the perspectives of both the patient and HCPs.^18^ Fundamentally, PCC meaning should include elements that go beyond following patients’ wishes by balancing patients’ preferences with contextual evidence, available resources and HCP considerations in a favourable interaction.

Mead and Bower’s PCC conceptualisation highlights practical elements as functions to be fulfilled during the patient-HCP encounter. These functions include the following: therapeutic patient-HCP relationship building, bio-psychosocial care provision, individualising the patient as a unique person, sharing power and responsibility and being realistic of the doctor as person.^12^ Whilst close to the epitome, this conceptualisation may not apply wholesale especially in health contexts that are paternalistic, with marked low patient medical literacy and limited human and therapeutic resources that may affect interactional behaviours.^19,20^

In chronic diseases, it is important to understand PCC interactions as they facilitate better care experiences and reported outcomes leading to patient empowerment and a proactive patient-HCP team.^21,22^ Some stated outcomes include patient satisfaction, perceived quality of care, patient adherence to management plans, reduced emergency visits, complications, patient empowerment and self-care and glycaemic control.^22,23^ In their meta-analysis of 109 correlational studies and 21 experimental studies, Haskard and Dimatteo found that HCP training on PCC was associated with 0.16 higher odds of patient adherence.^24^ It is vital to note that the evidence in literature is favourable to a patient-centred interaction in the management of chronic conditions such as DM. As we strive to advocate for PCC in chronic care encounters, it becomes imperative to gain its conceptual understanding. The study therefore sets out to understand the conceptualisation of PCC and highlight its functional elements in Malawi.

## Methodology

### Study design and setting

The study employed a qualitative exploratory approach. We collected data from four public hospitals’ DM clinics in the southern region of Malawi and the Ministry of Health headquarters. Because the public health sector is the biggest service HCP in Malawi, it was purposively chosen to highlight what PCC might look like in the sector. The four public hospitals included Queen Elizabeth Central Hospital (QECH), Zomba Central Hospital, Chikhwawa District Hospitals and Mulanje District Hospitals, and these were conveniently selected to highlight a mix of both urban and rural views. We selected policy makers at the Ministry of Health (MOH) headquarters, chosen from the Quality Management Directorate (QMD) and the Non-Communicable Disease and Injuries (NCDI) Directorate because of their influence in the development of chronic care policies.

### Study population and sampling

We interviewed 37 patients with DM, 33 HCPs and two policy makers. Healthcare providers encompassed nurses, clinical officers and medical doctors who are directly involved in DM care in defining the problem and rendering care.

The nurse in-charge purposively selected participants who were at least 18 years old, clinically stable and able to give a verbal and/or written consent to interviews and/or audio recording. Participants who could not give a written consent were excluded from the study.

### Data collection tools and procedures

The first author collected data using a semi-structured interview guide (Appendix 1). To encourage the participants to open up, we initially asked them to describe their typical patient-HCP interactions. We later zeroed in on to discuss their understanding of PCC.

The first author conducted individual in-depth interviews (IDIs) and focus group discussions (FGDs) to obtain both individual and collective meanings of PCC. One FGD was conducted at each facility and each FDG included between 10 and 11 participants. Patients and HCPs were in separate FGDs. Because of logistical challenges of getting enough policy makers together, no FGD was conducted with them. Focus group discussion participants’ selection followed the stated inclusion criteria.

We interviewed patients during their routine visit after obtaining care for that day, whilst clinicians were interviewed after their clinic duties. All interviews took place in secure rooms within the facility to maintain privacy. Data were collected from September 2019 to December 2019.

### Data management and analysis

Three research assistants translated the audio data from Chichewa into English and transcribed them verbatim. Later, we imported the data into NVivo software version 11.

The data analysis process started in the field by recording and exploring further on the themes that came out recurrently. Further analytical processes involved familiarisation, coding and mapping data against a Mead and Bower’s framework. Familiarisation with the data set was done through reading transcripts repetitively by the first author. Through this step, we identified the recurrent themes inductively from participants’ accounts until saturation. With input from the last author, the themes were grouped manually to come up with the initial codebook, which was iteratively refined through further discussion with the third and last authors. Exemplar extracts were then mapped against the codes.

We ensured triangulation of views presented in this study by collecting data from three participant categories, namely patients, their HCPs and policy makers. Moreover, member checking and the use of two data collection methods (FGDs and IDIs) allowed triangulation of individual and collective viewpoints. For quality assurance purposes, two randomly sampled transcripts were coded by the first and the third authors concurrently. The identified differences in coding were discussed and resolved. We achieved conformability by counterchecking the codes. The counterchecking process was done by an individual who was not in the study group to mitigate the effect of researchers’ prior predispositions about PCC.

## Findings

We conducted 31 individual participant IDIs (16 patients, 13 HCPs and two policy makers) and four FGDs. The distribution of the participants’ socio-demographic profile is shown in (Table 1).

**TABLE 1:** Participants’ socio-demographic characteristics.

Variable	Patients (*n* = 37)	Healthcare providers (*n* = 33)	Policy makers (*n* = 2)
**Age in years (average**)	50.4†	34.5‡	38.5§
**Sex**			
Male	12.0	22.0	2.0
Female	25.0	11.0	-
**Level of education**			
None	10.0	-	-
Primary	7.0	-	-
Secondary	10.0	-	-
Tertiary	10.0	33.0	2.0
**Type of DM**			
Type 1	3.0	-	-
Type 2	34.0	-	-
**Duration DM (years)**			
< 2	7.0	-	-
2–5	10.0	-	-
> 5	14.0	-	-
**Current facility use (years)**			**-**
< 2	5.0	-	-
2–5	21.0	-	-
> 5	5.0	-	-
**Healthcare provider cadre**			
Nurses	-	6.0	-
Clinical officers	-	19.0	-
Medical officers	-	5.0	-
Specialist doctors	-	3.0	-
**Healthcare provider service (years)**			
< 2	-	9.0	-
2–5	-	6.0	-
> 5	-	7.0	2.0

DM, diabetes mellitus.

Average (years) s.d.:

† , Patients = 13.1;

‡ , Healthcare providers = 11.5;

§ , Policy makers = 1.4.

### The general conceptualisation of patient-centred care

Regarding the general understanding of PCC, the HCPs seemed to express some familiarity with the term and offered meanings similar to what literature offers. Conversely, some patients expressed difficulty in understanding PCC and, consequently, attempted to simplify the term in their own understanding and provided alternative terms. This is exemplified by the following participant response:

‘… I think you asked that how do I see that I have been satisfied with the care we receive here once we have entered into the hospital premises.’ (37-year-old, urban-based, female patient)

Not all conceptualisations were presented explicitly as meanings but also as experiences and expectations of care. Furthermore, the participants stated that PCC is the care that satisfies the patient, brings happy emotions and signifies government’s commitment to ‘best care’ delivery. In that regard, one participant observed the following:

‘In my opinion, I think the government wants the patient to be centred. An evidence of that the government wants the patient to be centred is that 3 weeks ago I was also selected to be part of a certain research. So that research is trying to find out the exact drugs meant for Africans. So, on that I think the government is very much interested to give the best care to its people.’ (62 year-old, urban-based, male patient)

The emergent themes and their functional elements are summarised in Table 2. It is noted that most of the themes were validated by all stakeholder groups with minor expression differences. To highlight the study findings’ comparability with prior works, the results are mapped against Mead and Bower’s framework.^12^

**TABLE 2:** Qualitative elements of patient-centred care by patients, healthcare providers and policy makers.

PCC functional element	The study’s conceptualisation of PCC		
	Patients	Healthcare providers	Policy makers
Bio-psychosocial approach	Adequate equipment for through bodily exam.	Bio-psychosocial approach. All health problems addressed. Team approach including patient.	Bio-psychosocial care.
Individualising care to the patient as a unique person	-	Addressing unique needs and expectations.	Achieving biomedical goals.
Sharing power and responsibility	Being consulted on care decisions. Leveraging patients’ capacities.	Active participation in- and empowering patients to make decisions. Sharing responsibilities.	Involve patients in design of health care services. Shared decision making.
Enhancing the patient-doctor relationship	Respect, dignity, friendliness, being listened to. Allowing time to talk	Respect, dignity, being listened to. friendliness.	Respect, dignity.
The doctors as person: Being realistic	Patients acknowledging the HCP/system has challenges to care delivery.	Recognise contextual limitations to care delivery.	Recognise contextual limitations to care delivery.
	Medication availability and access.	Essential medication availability access.	Better medicine supply chain.
	Timeliness of care.	Timeliness of care.	-
	Informing, educating, support, feedback, validation, reprimand.	Educating patients for informed choices.	-

HCP, healthcare provider; PCC, patient-centred care.

### Meeting individualised needs, goals and expectations

The healthcare workers reported that PCC meant tailoring care with unique individual needs, life circumstances and expectations. In that vein, one participant observed the following:

‘I think each and every patient has his/her own expectations when coming to the hospital. As a result, when delivering the care to the patient, we have to deliver in a way that we will be able to meet the patients’ expectations and that will be patient centred.’ (63-year-old, urban-based, female, nurse)

From the perspective of the HCPs, meeting biomedical goals of care, such as glycaemic control, can be considered as being patient centred, as observed by another HCP:

‘So, if we have assessed that a patient is doing good in terms of his diabetes or hypertension, I think that can mean patient centred care.’ (29-year-old, urban-based, male)

### Accessing diabetes medication

Both patients and policy makers conceptualise PCC as receiving medication. This was a prominent theme as one patient participant explains:

‘[PCC] … It’s what I have already explained. I should leave here with all the drugs that have been prescribed for me, that way I would feel so happy.’ (58-year-old, rural-based, female patient)

Accessing medication seems to be the gauge for PCC. When the senior policy maker is asked about his vision for PCC, he emphasised the following:

‘I’ll be happy to see a patient coming to the facility for a refill and drugs are available. That could be ideal because […]. I’d be happy if our supply chain is up to date.’ (40 year-old, MOH, male, policy-maker)

The availability and access to adequate medicines were regarded as a basic need, expectation and requirement for appraising a PCC encounter.

### Supporting relational aspects of care

Both patients and HCPs conceptualise PCC as relationship building, stating it as a process of care, that should start with a warm welcome and being listened to with friendly and dignifying conversations. More desired elements of PCC encounters are presented as friendliness, dignity, respect and the opportunity for patients to express themselves. These are, in part, reflected in the following verbatim responses:

‘We should have a peaceful and amicable conversation. […] If you have done something wrong, you should be told in an amicable manner what you were supposed to do with dignity.’ (18-year-old, urban-based, male patient)

‘… I feel like patients need to feel like we are interested to talk with them […] by actions and words.’ (27 year old, rural-based, male HCP, clinical officer)

Harsh and hurried encounters are reported by the patients. Again, the patients highlight that warm receptions and friendly environments can create trust and openness that facilitate the building of therapeutic relationships even in the face of limited therapeutic resources.

Given the relational power dynamics that exist during patient encounters, the HCPs recommend that relationship building, recognition and minimising the power gap should be their initiative using simple and socially appropriate strategies. In that regard, one participant observed the following:

‘… I was telling, just two days ago, [*t*]he colleagues hear simple concepts on how to put chairs in consultation rooms. Put the chairs in a way that you tell the patient that I am your friend […]. So that is something very simple, the way you are going to set your encounter, furniture so to establish that friendship.’ (50-year-old, urban-based, male, HCP)

### Patient involvement in care

Although expressed differently between patients and HCPs, the theme depicts a spectrum of tasks in shared responsibility and decision making, from a mere assent from patients to empowering patients with information for decision making. The patients expressed the desire to be consulted before care decisions are made, as illustrated in the following verbatim response:

‘… But just making decisions without the knowledge of the patient, sometimes it becomes a problem.’ (38 year-old, urban-based, female, patient)

Even though the patients wish to be involved in major care decisions, they perceive difficulty and inappropriateness to debate or reason with the HCP. Most passively accept the proposed care based on the understanding that their HCP knows it all. This is, in part, reflected in the following verbatim response:

‘My suggestions, if you are [*a*] patient and you are meeting the doctor, there is no way you can be exchanging ideas. The doctor just tell[*s*] you that depending on your results, follow this this and this […]. I do not think a patient can have the audacity to be telling the doctor what to do as if you know about health. You just listen and take whatever the doctor has told you.’ (48-year-old, urban-based, male, patient)

In contrast, and for HCPs and policy makers, PCC goes beyond assent and involves the patient from designing the need-based services to negotiating care choices with mutuality. The HCPs agree that patients should be active recipients of care and their capabilities leveraged, as observed in the following participant response:

‘So, I am saying that patient-centred care is all about putting the patient at the centre of the care. […] So when you are designing systems, or programmes, the programmes should be based on the needs of the patient, not making your patient follow the system or making the patient to be a passive recipient of care but should be in a position that is driving the care to them.’ (37 year-old, MOH, male, policy-maker)

### Informing, educating and counselling

This theme was very prominent both for HCPs and patients. It captures that PCC encounters should be an opportunity for information sharing. The information could be shared through counselling and when educating patients about disease, its treatment and prognosis and the expectations of care. In light of this, a patient observed the following:

‘When they counsel us on which foods to eat and not to eat … they counsel us on marriages because, sometimes, there is a decrease in libido. […] Also, as diabetic patients, we are advised bathe every day because we develop a very pungent odour due to the diabetes.’ (60-year-old, rural-based, female, patient)

Furthermore, emphasising PCC as an information-sharing function, it empowers patients to make informed choices, as reflected by one HCP:

‘So, patient-centred [*care*] now will be, after giving the patient such information, what will she choose? What would she feel that is better or feasible, practical to more adapt to her lifestyle, or whatever is linked or concerns her, but based on, what you have shared with her, so I think that is what I would feel about patient-centred[*ness*].’ **(**35-year-old, urban-based, male, HCP)

Information sharing is further depicted to serve as support towards self-care through giving hope, feedback, reprimand and validation. In that vein, one patient had this to say:

‘You should tell us the procedure on how we are to take medicine because, sometimes, we forget […] from there they should give us feedback on how we are progressing. Is our diabetes condition improving or not? […] Because it is like you give hope to patients.’ (65-year-old, rural-based, male, patient)

### Receiving wholesome care

Both patients and HCPs alluded to PCC being wholesome, and that reflects care that thoroughly assesses bio-psychosocial aspects of life, as recollected by one participant:

‘Patient centred means a holistic approach to the patient. Once you have approached him/her holistically, it means it is part of the patient centred […]. It means we are seeing the patient physically, mentally, as well as spiritually.’ (27-year-old, rural-based, male, HCP)

This view was shared by another participant who recollected the following:

‘In my opinion, I think it [*PCC*] means patients must receive the necessary and holistic care […]. So, when they talk of patient-centred care, the care must be wholesome […] and be thoroughly examined.’ (47-year-old, rural-based, female, patient)

The participant verbatim responses further highlight PCC as a comprehensive assessment and management of patients’ problems over one or several encounters. Wholesomeness also extended to include managing patients throughout their illness by a team that includes the patient, as reflected in the following response:

‘Patient-centred care is basically the total care given to a patient as a team. Everyone has a part to play. Be it doctors, nurses, patients as well as guardians.’ (34-year-old, urban-based, male, HCP)

### Timeliness of care

Untimely care was expressed as an expected yet failed element of PCC amongst patients and HCPs. Timeliness referred to how urgent care is delivered and the length of time patients spend at the clinic. This is, in part, reflected in the following participant response:

‘Sometimes the reception we receive is not good. A patient might come here in the morning without being attended to until evening, and that disappoints us. We should properly be received and the HCPs should attend to us […]. What we want is punctuality.’ (18-year-old, urban-based, male, patient)

Moreover, the HCPs also expressed the awareness that delays in service provision affects the patients’ perception of patient centredness. In that regard, one participant observed the following:

‘… Even sometimes they [*patients*] become angry because they have been waiting for a longer time and for the conversation to be OK, it becomes very difficult because they feel like they are not being taken care of because […] the clinic started late.’ (25-year-old, urban-based, male, nurse)

### Being realistic and pragmatic

Most HCPs related their conceptualisation of PCC with realism, further articulating their frustration with the systemic constraints, as illustrated in the following verbatim response:

‘We want the care to be patient-centred such that we want to make plans with the patient, but it has not possible because of human resources. Sometimes, HCPs look at the queue that is outside, maybe 40 to 50 patients. I think that is why we are failing because we want to finish the line but not necessarily having enough time to manage the patients.’ (40-year-old, MOH, male, policy-maker)

Whilst acknowledging the challenges in realising PCC, the HCPs suggested some simple and practical tips for improvement.

## Discussion

This article discusses the functional elements in the conceptualisation of PCC, its similarities with prior frameworks and the peculiarities to this context. The centrality of meeting patients’ needs and expectations of care, and relationship building, is highlighted. Furthermore, the study reveals how patients and HCPs perceive some failed elements of PCC and their realistic approach in the face of limited human and therapeutic resources. Based on the collective meanings, this article proposes a working definition.

We conceptualise PCC as meeting individual patients’ needs, goals and expectations, especially as it relates to health system responsiveness.^22,25,26^ This is consistent with Tolib et al.’s systematic review of literature, in which the authors noted how receptivity to patients’ needs and expectations was important to appraise quality from patients’ perspectives.^26^ The patients, in our study, expressed receptivity by narrating or rating experiences of their care. Individual patient needs included supportive diabetic education, amicable relationships and glycaemic control, whilst expectations consisted of access to medication. Overall, we found that measuring experiences of the interaction may be a proxy for measuring the quality of PCC.

Receiving DM medication is an emphasised and peculiar element in this context. Even though it falls outside the conventional patient-HCP interactional-based definition, it is not surprising. The emphasis is likely because medication is a basic need for every patient with DM and, therefore, the most sought-after aspect and gauge for appraising PCC.^27,28^ More so in this resource-constrained setting like Malawi, the prominence of this element is heightened by medication shortages that are prevalent.^29^ Functionally, the finding highlights the centrality of meeting the basic patient needs, be it technical or interpersonal.

Even in the face of limited medicinal resources, the patients emphasise that relationship building is a valued element in PCC encounters over infrastructure and doctors’ knowledge.^22^ The positive role of the perceived quality of relational dynamics in DM patient-HCP interaction is confirmed.^30,31,32^ A multi-country Diabetes Attitudes, Wishes and Needs (DAWN) study reported a positive association between better patient-HCPs relationship and patient outcomes.^33^ Specifically, the emotional elements of the unrushed encounters based on HCP attentiveness, warmth, empathy, trust and opportunity to respond to patient’s questions were highly associated with reduced diabetes-related distress and hyperglycaemic symptoms, better glycaemic control and general well-being, lifestyle and medical regimen adherence and perceived diabetes control.^33^ This confirms the proposition that investments made in relationship building can improve health-seeking behaviours and patient outcomes.

Patients’ active involvement in care is a desired element of PCC amongst patients with DM.^34,35,36^ Whilst HCPs conceptualise active involvement from design to delivery of care, patients largely experience assent. This is partly explained by the difference in how patients and HCPs approach the interaction in this context. Because of the social and power gaps between patients and HCPs,^37,38^ patients often approach the interaction from an uninformed and powerless perspective,^39^ and this reduces their propensity to actively participate. Again, whilst HCPs report patients’ involvement with normative standards, but, in practice, they often make contextually negotiated decisions as the extent to which patients can be involved.^40^ Consequently, patients’ experience and perception of involvement are often limited to assent and being grateful recipients of care.

Like other frameworks, information sharing is an important element and serves communicative roles such as informing, educating, counselling, reprimanding, validating, supporting and empowering patients to make informed choices.^41,42,43^ Although most of these facets of information sharing are important, the HCPs, in our study, uniquely identify that PCC is about information that empowers the patient to choose what is ideal for self-care. Mulder et al. confirm the role of transformative education as a basic tenet of diabetic PCC.^43^ Mulder et al. point out that transformative education empowers patients to be active participants within the provisions of PCC.

Holistic care in this study encompassed attending to bio-psychosocial aspects of patients’ life throughout the illness journey. It also included holistic management of the patients’ bio-psychosocial aspects by a multidisciplinary team that includes the patients themselves. Holism is validated as a co-ordinated care that addresses patients’ needs at the juncture of the bio-medical ‘pathology’, the person experiencing the pathology, their context (bio-psychosocial) and the nature of the interaction between the person and their context.^44^ Even though holism sounds complex, the acknowledgement of the patient as an active partner in the team is commendable in this context.

Untimely care is perceived as a failed element of PCC. Timeliness of care gives the perception of better healthcare care organisation and access that is core to PCC experience.^45^ Even though WHO highlights timeliness of care as a distinct element of quality, understandably, the participants, in this study, pointed out to long waiting times as undermining efforts towards PCC. This, therefore, needs to be addressed.

Being realistic is a crucial contextual element in PCC operationalisation. It is expressed as the frustration of the HCPs of knowing the elements of PCC, yet being unable to practise, or employing workaround behaviours and alliances in order to be pragmatic. From the perspective of an optimist, it is a sign of acknowledgement of shortcomings and positive attempts towards advancement of PCC in the face of systemic challenges.^46^ Contextual challenges purportedly explain the employment of workaround strategies and the redefinition of PCC to what is practically possible.^40^ Thus, as we continue to advocate for government’s commitment towards conducive environment for PCC, we ought to view contextual moderations as a crucial element of any PCC conceptualisation.

### Limitations of the study

This study obtained insights from one single chronic disease that may not be representative. However, it was envisaged that patients with DM’s repetitive contact in a non-acute setting gathers important elements around PCC conceptualisation. Because of limited time, we did not do ethnographic observations to examine the individual patient-HCP interactional dynamics that could have enriched our findings. Finally, owing to the paucity of PCC frameworks in Africa, there is a possibility that the Western-oriented analytical framework, which guided our analysis, might have created a Western inclination in interpreting the results.

## Conclusion

This study confirms the complexity and interrelatedness of PCC elements. In the Malawi[-an] context, we conceptually understand PCC as an expected care process that incorporates warm patient reception, where the HCP consciously aims to reduce the patient-HCP power gap to harness a good long-term relationship. This creates a conducive atmosphere that allows gathering of information that holistically identifies the individual specific problems and all possible interacting factors, ensuring timely access to care and medication.

The functional elements of PCC are, in many ways, similar to those in literature. However, its expressions go beyond the patient-HCP interactional realm to include the wider organisation themes such as access to medication, amongst others. We expect that these findings will contribute to the actionable dialogue around PCC to clearly communicate its transformative vision.

### Recommendations

Whilst Malawi is striving to be patient centred in its chronic care delivery, it needs to address the wider organisational issues in the work environment and the supply chain to realise PCC practice. The HCPs’ pre-service education must include skills in relationship building and information sharing that essentially empower the patient to demand and participate in PCC.

## Appendix 1: Semi-structured data collection tools for in-depth interviews (patients with diabetes mellitus, healthcare providers and policy makers) and focus group discussions

### 1a: Semi structured DM patient interview guide

‘Probes for Patients with DM’ In-depth Interviews and focus group discussion which are subject to adaptation from the scoping review findings’

- Please tell me about the care that you received at this clinic recently.
  ■ Please explain in detail about the kind of interaction had between you and your HCP.
  ■ Tell me about the expectations of the care you want to receive at this clinic.
  ■ If you were to share with somebody your experiences about the care you received at this clinic, what would you say?
- For the care to be ‘centred on you’, what does that entail to you? What should happen during the interaction with your HCP to make such care ‘centred on you’, What key elements of the interaction with your HCP would make such care ‘centred on you?’ (including cues that point to some important elements of PCC) or (use case vignettes of some PCC dimensions such as shared decision making)
- As an example, do you have an opportunity to sit down to find a consensus of what should be done about your condition?
  ■ Please explain; how did it go?
  ■ Use of hypothetical scenarios such as the following: ‘I would like to give you a scenario. Maybe you have a high blood sugar, and you have ideas on how best you can manage the condition whilst your doctor has a contrary idea. How would such an interaction go’?
- Please tell me about the practice of care that is ‘centred on you’ amongst HCWs at this facility:
  ■ In your opinion, what do you think are the factors (both positive and negative) that are influencing the type of patient-HCP interactions that you experience?
  ■ What do you think are the enabling factors for the HCWs to render care that is deemed to be ‘centred on you’?
  ■ (In case HCPs already do provide care centred on you) What do you think would help reinforce the interactions that you have at this facility?
- Do you have any questions?

### 1b: Semi structured interview guide for in-depth interviews for HCWs

‘Probes for HCWs in-depth interviews and focus group discussion which are subject to adaptation from the scoping review findings’

- Tell me about the care you render to the patients that come to this clinic.
  ■ How do you go about interacting with the patients that receive care at this facility?
  ■ Can you describe to me a typical interaction with a patient?
- Tell me about the care that you think your patients would like to receive.
  ■ What do you think are the expectations of your patients in your interaction with them?
- In your opinion, if the interaction is to be described as patient centred, what would it entail?
- For the care to be ‘centred on the patient’, what does that entail, What should happen during the interaction with your patient to make such care ‘centred on them’, What key elements of the interaction would make such care ‘centred on the patient?’ (including cues that point to some important elements of PCC) or (use case vignettes of some PCC dimensions such as shared decision making)
- As an example, do you have an opportunity to sit down to find consensus with a patient?
  ■ Please explain; how do you go about it?
  ■ Use of hypothetical scenarios such as the following: ‘I would like to give you a scenario. Maybe your patients have a high blood sugar, and they have ideas on how best to manage their condition, whilst you as an HCP has a contrary idea. How would such an interaction go’?
- In your opinion what do you think are the factors (Both positive and negative) influencing the type of interactions that you experience?
- What do you think are the enabling factors for you to render care that is deemed to be ‘centred on the patient’?
- (In case HCPs already do provide care that centres on patients) What do you think would help reinforce the interactions that you have at this facility?
- Tell me about what would it take to practise care that is centred on the patient.
  ■ What would motivate you to render care and interactions that focus on the patient? ‘(An example of motivation; what is the value of care that centres on patients in your care delivery if any? How do you think it contributes to the goals of quality care?)’
  ■ What do you think is required for you to be able to render care that focusses on the patient? ‘(An example: what specific information, skills and attitudes do you require to render care that is centred on patients?)’
    ■ Do you feel empowered to render care that is centred on your patients (self-efficacy)? (How and from where do you get the empowerment; If not empowered, why?; What is your opinion of the power differences between the implements (HCWs & policy makers) and patients in the implementation of care that centres on patients?)
- Further questions as necessary. Do you have any questions?

### 1c: Semi structured interview guide for in-depth interviews for policy makers

‘Probes for healthcare workers (HCWs) in-depth interviews which are subject to adaptation from the scoping review findings’

- Tell me about the care that is rendered to the patients in this clinic.
  ■ How do you go about interacting with the patients that receive care at this facility?
  ■ In your opinion, can you describe to me a typical interaction with a patient ought to look like?
- Tell me about the care that you think your patients would like to receive.
  ■ What do you think are the expectations of your patients with your HCWs (including yourself) interaction with them?
- In your opinion, if the interaction is to be described as patient centred, what would it entail?
- In your opinion what do you think are the factors (Both positive and negative) influencing the type of interactions that you experience?
- What do you think are the enabling factors for the HCWs to render care that is deemed to be ‘centred on the patient’?
- (In case HCPs already do provide care that centres on patients) What do you think would help reinforce the interactions that you have at this facility?
- Tell me about what would it take to improve the care practice that is centred on the patient.
  ■ What would be the motivations to render care and interactions that focus on the patient?
    ‘(An example of motivation; what is the value of care that centres on patients in your care delivery if any? How do you think it contributes to the goals of quality care?)’
  ■ What do you think is required for HCWs to be able to render care that focusses on the patients at the facility?
    ‘(An example: what specific information, skills and attitudes do you require to render care that is centred on patients?)’
- Do you think the HCWs feel empowered to render care that is centred on patients?
  ■ How and from where do you get the empowerment?
  ■ If not empowered, why?
  ■ What is your opinion of the power differences between the implements (HCWs & policy makers) and patients in the implementation of care that centres on patients?
- In your opinion, what are the policy recommendations that are required to facilitate rendering care that is centred on the patient?
- Further questions as necessary.
- Do you have any questions?

### 1d: Focus group discussion probes for patients with DM

- For the care to be ‘centred on you’, what does that entail to you? What should happen during the interaction with the HCP to make such care ‘centred on you’,
- What key elements of the interaction with your HCP would make such care ‘centred on you?’ (Including cues that point to some important elements of PCC obtained from the scoping review) or (use case vignettes of some PCC dimensions)
- What do you think helps to deliver such care? Or make such interactions centred on the patients views

What do you think hinders you from receiving such care?

### 1e: Focus group discussion probes for healthcare workers

- For the care to be ‘patient or person centred’, what does that entail to you? What should happen during the interaction with the patient/ or client that would make such care ‘centred on them?’ (Including cues that point to some important elements of PCC obtained from the scoping review) or (use case vignettes of some PCC dimensions)
- What do you think helps to deliver such care? Or make such interactions centred on the patients views
- What do you think hinders you from delivering such care?
- Do you have anything else to add?
